# Clinical Feasibility of Six Inflammatory Markers for Predicting the Mortality of Patients With Cancer: Longitudinal Study

**DOI:** 10.2196/99410

**Published:** 2026-07-22

**Authors:** Kangwei Wang, Ce Shi, Dingchao Xia, Ya Lin

**Affiliations:** 1 Department of Cardiology, Affiliated Yueqing Hospital of Wenzhou Medical University, Wenzhou, China; 2 School of Digital Economics, Wenzhou Vocational College of Science and Technology, Wenzhou, China; 3 Department of Infectious Diseases, Wenzhou Central Hospital, Wenzhou, China; 4 Department of Infectious Diseases, Wenzhou Sixth People's Hospital, Wenzhou, China; 5 Wenzhou Key Laboratory of Diagnosis and Treatment of Emerging and Recurrent Infectious Diseases, Wenzhou, China; 6 MAFLD Research Center, Department of Hepatology, The First Affiliated Hospital of Wenzhou Medical University, Nanbaixiang Street, Ouhai District, Wenzhou, 325000, China, 86 15057503329

**Keywords:** inflammatory markers, all-cause mortality, cancer mortality, cardiovascular mortality, prognosis

## Abstract

**Background:**

Emerging evidence indicates that inflammation plays a crucial role in cancer prognosis. Inflammatory response biomarkers are recognized as promising prognostic factors for mortality in patients with cancer.

**Objective:**

This study aims to evaluate the prognostic significance of the systemic inflammatory response index (SIRI), systemic immune-inflammation index (SII), platelet-to-lymphocyte ratio (PLR), neutrophil-to-lymphocyte ratio (NLR), inflammatory prognostic index (IPI), and C-reactive protein-albumin-lymphocyte (CALLY) index.

**Methods:**

Weighted Cox regression analyses, restricted cubic spline models, Kaplan-Meier survival curves, and receiver operating characteristic analyses were performed to assess the predictive value of the 6 inflammatory markers for mortality. Subgroup analyses and sensitivity analyses were conducted to examine associations within specific subpopulations.

**Results:**

Cox regression models demonstrated that SIRI, NLR, IPI, and CALLY were significant predictors of all-cause mortality (tertile 3 vs tertile 1; hazard ratio [HR]: SIRI: 1.72, 95% CI 1.29‐2.27; NLR: 1.33, 95% CI 1.02‐1.74; IPI: 1.48, 95% CI 1.14‐1.92; CALLY: 0.66, 95% CI 0.51‐0.85). IPI (HR 1.91, 95% CI 1.11‐3.27) and CALLY (HR 0.53, 95% CI 0.31‐0.90) were significantly associated with cancer-specific mortality, whereas only SIRI was able to predict cardiovascular mortality (*P* value for trend=.04). Dose-response relationships were observed between the 6 inflammatory markers and mortality outcomes. Kaplan-Meier survival curves further illustrated significant differences between tertile groups (log-rank test, *P*<.001). The 6 inflammatory indices exhibited moderate predictive ability for all-cause mortality. IPI yielded the highest area under the curve (AUC) for cancer-specific mortality (AUC=0.6338), and SIRI was the most efficient predictor of cardiovascular mortality (AUC=0.687). No significant interactions were observed between the 6 inflammatory markers and most subgroup variables.

**Conclusions:**

SIRI, NLR, IPI, and CALLY represent convenient and cost-effective prognostic tools for predicting mortality in patients with cancer. In contrast, SII and PLR may not be reliable prognostic biomarkers.

## Introduction

Cancer remains a major societal, public health, and economic burden, ranking as the third leading cause of death worldwide [1-3]. Although advances in diagnosis and treatment have substantially reduced cancer-related mortality rates, the number of cancer survivors is projected to exceed 26 million by 2040 [4,5]. Nevertheless, cancer treatments—particularly anticancer agents—may induce cardiotoxicity and elevate the risk of cardiovascular disease (CVD) [6]. Moreover, cancer and CVD may share common pathophysiological pathways, given the overlapping risk factors, inflammatory processes, metabolic dysregulation, and neurohormonal activation associated with both conditions [7,8]. Consequently, there is an urgent clinical need for early detection and routine screening of all-cause, cancer-specific, and cardiovascular mortality among patients with cancer, using simple, cost-effective, and readily accessible tools.

Accumulating evidence has established inflammation as a common pathogenic driver of various diseases. Inflammation is a hallmark of tumorigenesis and plays a critical role in cancer initiation, progression, and metastasis [9-11]. Similarly, CVD is recognized as a chronic inflammatory condition, with inflammation being central to the onset and progression of atherosclerosis [11-13]. Inflammatory status can be assessed using various immune cell parameters and biochemical markers. Furthermore, systemic inflammatory responses contribute to decreased serum albumin levels. Wu et al [14] demonstrated that albumin levels were associated with cause-specific mortality in older adults [14]. In recent years, novel composite inflammatory markers—including the systemic inflammatory response index (SIRI), systemic immune-inflammation index (SII), platelet-to-lymphocyte ratio (PLR), neutrophil-to-lymphocyte ratio (NLR), inflammatory prognostic index (IPI), and C-reactive protein-albumin-lymphocyte (CALLY) index—which integrate complete blood count (CBC) with albumin and C-reactive protein (CRP)—have emerged as promising prognostic indicators for predicting disease outcomes [15-19].

The primary objective of this study was to evaluate the predictive value of SIRI, SII, PLR, NLR, IPI, and CALLY for all-cause, cancer-specific, and cardiovascular mortality in patients with cancer, and to further explore the potential use of these markers in the early identification of mortality risk.

## Methods

### Study Population and Exclusion Criteria

Data for this study were obtained from the National Health and Nutrition Examination Survey (NHANES) [20], a program designed to generate vital and health statistics for the US population. Given that CRP data were available only for 5 consecutive survey cycles (2001‐2002, 2003‐2004, 2005‐2006, 2007‐2008, and 2009‐2010), we initially collected data from 52,195 participants across these cycles. Participants were excluded if they met any of the following criteria: (1) missing blood test results for albumin, CBC, or CRP; (2) absence of a cancer diagnosis or missing information on cancer history; (3) missing data on relevant covariates; or (4) pregnancy.

### Evaluation of 6 Inflammatory Markers

For all participants, CBC was performed on blood specimens using a Beckman Coulter MAXM instrument at the Mobile Examination Centers. CRP levels were quantified using latex-enhanced nephelometry, and albumin concentrations were measured using the DcX800 method.

The inflammatory markers were calculated using the following formulas:


$$SIRI=\frac{neutrophil\;count\;\times \;monocyte\;count}{lymphocyte\;count}$$

$$SII=\frac{neutrophil\;count\;\times \;platelet\;count}{lymphocyte\;count}$$

$$PLR=\frac{platelet\;count}{lymphocyte\;count}$$

$$NLR=\frac{neutrophil\;count}{lymphocyte\;count}$$

$$IPI=\frac{CRP\;\times \;NLR}{albumin}$$

$$CALLY=\frac{albumin\;\times \;lymphocyte\;count}{CRP\;\times \;10}$$

### Outcome and Follow-Up

Cancer diagnosis was based on self-reported medical history. Participants were identified as having cancer if they responded affirmatively to the question: "Have you ever been told by a doctor or other health professional that you had cancer or a malignancy of any kind?” Mortality status for the NHANES follow-up population was obtained from the publicly available mortality files [21]. The primary outcome was all-cause mortality, while secondary outcomes included cancer-specific and cardiovascular mortality, coded according to the *International Classification of Diseases, 10th Revision* (*ICD-10*). Follow-up time was calculated in person-months from the date of the interview, with the observation period ending on December 31, 2019.

### Assessment of Covariates

Demographic characteristics included gender, age, race, educational level, marital status, smoking status, alcohol consumption, and the poverty-to-income ratio. Race was categorized as non-Hispanic White, non-Hispanic Black, other Hispanic, Mexican American, or other races. Educational level was classified as: less than 9th grade, 9th to 11th grade (including 12th grade with no diploma), high school graduate or General Educational Development diploma, some college or associate degree, and college graduate or above. Marital status included the following categories: married, widowed, divorced, separated, never married, and living with a partner. Participants who consumed at least 12 drinks of any type of alcoholic beverage within 1 year were defined as drinkers. Those who reported smoking at least 100 cigarettes during their lifetime were categorized as smokers. Medical history—including hypertension, diabetes, hyperlipidemia, myocardial infarction (MI), congestive heart failure (CHF), coronary heart disease (CHD), and stroke—was based on prior medical records provided by health care professionals or physicians.

### Statistical Analysis

The distribution of continuous variables was assessed using the Shapiro-Wilk test. Given the nonnormal distribution of the variables, they were summarized as medians with IQRs. Categorical data were expressed as frequencies and percentages. Differences among groups stratified by the 6 inflammatory markers were evaluated using the Kruskal-Wallis test or Fisher exact test, as appropriate.

To evaluate the prognostic value of the 6 inflammatory markers for mortality, weighted Cox regression analyses were performed to estimate hazard ratios (HRs) with 95% CIs. Three adjustment models were constructed: model I was unadjusted; model II was adjusted for gender, age, race, educational level, marital status, and poverty-to-income ratio; and model III was additionally adjusted for smoking, alcohol consumption, hypertension, diabetes, hyperlipidemia, CHF, CHD, MI, and stroke, in addition to the covariates included in model II. Restricted cubic spline (RCS) models were further used to explore potential dose-response relationships between the inflammatory markers and mortality outcomes, with adjustment for demographic characteristics, smoking, alcohol consumption, and disease history. Kaplan-Meier survival curves with log-rank tests were used to estimate survival distributions across tertiles of the inflammatory markers. Receiver operating characteristic curves were generated to assess the sensitivity and specificity of the 6 inflammatory markers, and the area under the curve (AUC) was calculated to evaluate their predictive performance for all-cause, cancer-specific, and cardiovascular mortality. Subgroup analyses were conducted concurrently to elucidate potential interactions between the 6 inflammatory markers and covariates. To assess the robustness of the results, a sensitivity analysis was performed excluding patients with asthma and arthritis.

All statistical analyses were performed using R software (version 4.3.0; R Core Team 2023), and a *P* value of less than .05 was deemed statistically significant.

### Ethical Considerations

The protocol for NHANES was approved by the National Center for Health Statistics Ethics Review Board (protocols: 98‐12 and 2005‐06 [22]) in accordance with the Declaration of Helsinki. All participants signed written informed consent.

## Results

### Baseline Characteristics

A total of 946 participants were enrolled in this study (Figure S1 in Multimedia Appendix 1). Based on all-cause mortality status, participants were classified into survival and nonsurvival groups. Table 1 summarizes the weighted baseline characteristics comparing the 2 groups. The survival group had a higher proportion of women, was younger, and exhibited higher educational levels, higher income, and more favorable marital status compared with the nonsurvival group. The nonsurvival group reported a higher prevalence of hypertension, diabetes, CHF, CHD, MI, stroke, and weak or failing kidneys. Furthermore, the values of SIRI, SII, PLR, NLR, and IPI were higher in the nonsurvival group than in the survival group, whereas the CALLY index was lower in the nonsurvival group (Table 1; Figure S2 in Multimedia Appendix 1).

**Table 1.** Survey-weighted baseline characteristics of individuals from the National Health and Nutrition Examination Survey.

Characteristics	Overall (weighted N=7,374,727.2)	Survival (weighted n=4,573,558.9)	Nonsurvival (weighted n=2,801,168.3)	*P* value
Gender, n (%)				<.001
Man	3,047,010.5 (41.3)	1,641,241.2 (35.9)	1,405,769.3 (50.2)	
Woman	4,327,716.7 (58.7)	2,932,317.7 (64.1)	1,395,399.0 (49.8)	
Age (y), median (IQR)	63.00 (52.00-74.00)	58.00 (48.00-67.00)	74.00 (65.00-80.00)	<.001
Race, n (%)				.12
Mexican American	132,737.2 (1.8)	99,944.5 (2.2)	32,792.8 (1.2)	
Other Hispanic	150,294.8 (2.0)	52,640.9 (1.2)	97,653.9 (3.5)	
Non-Hispanic White	6,633,329.9 (89.9)	4,147,593.8 (90.7)	2,485,736.1 (88.7)	
Non-Hispanic Black	328,374.8 (4.5)	166,095.2 (3.6)	162,279.5 (5.8)	
Other race, including multiracial	129,990.5 (1.8)	107,284.5 (2.3)	22,706.1 (0.8)	
Educational level, n (%)				<.001
Less than 9th grade	487,769.1 (6.6)	154,587.5 (3.4)	333,181.6 (11.9)	
9-11th grade (includes 12th grade with no diploma)	845,599.6 (11.5)	403,359.8 (8.8)	442,239.8 (15.8)	
High school graduate or general equivalent diploma	1,882,405.5 (25.5)	1,110,510.7 (24.3)	771,894.8 (27.6)	
Some college or associate’s degree	2,026,301.5 (27.5)	1,304,406.2 (28.5)	721,895.3 (25.8)	
College graduate or above	2,132,651.5 (28.9)	1,600,694.8 (35.0)	531,956.7 (19.0)	
Marital status, n (%)				<.001
Married	4,831,440.5 (65.5)	3,250,681.2 (71.1)	1,580,759.3 (56.4)	
Widowed	999,693.0 (13.6)	324,868.2 (7.1)	674,824.7 (24.1)	
Divorced	853,298.7 (11.6)	567,372.8 (12.4)	285,925.9 (10.2)	
Separated	137,531.6 (1.9)	91,109.7 (2.0)	46,421.9 (1.7)	
Never married	348,198.2 (4.7)	220243.0 (4.8)	127,955.2 (4.6)	
Living with partner	204,565.3 (2.8)	119,284.0 (2.6)	85,281.3 (3.0)	
PIR^a^, median (IQR)	3.32 (1.76-5.00)	4.03 (2.18-5.00)	2.24 (1.33-3.79)	<.001
Smoking, n (%)				.12
Yes	4,333,336.8 (58.8)	2,596,368.8 (56.8)	1,736,968.1 (62.0)	
No	3,041,390.4 (41.2)	1,977,190.2 (43.2)	1,064,200.2 (38.0)	
Drinking, n (%)				.06
Yes	5,063,639.4 (68.7)	3,260,109.2 (71.3)	1,803,530.3 (64.4)	
No	2,311,087.8 (31.3)	1,313,449.7 (28.7)	997,638.0 (35.6)	
BMI (kg/m^2^), median (IQR)	27.47 (23.95-31.96)	27.49 (23.98-32.07)	27.24 (23.84-31.64)	.77
Waist circumference (cm), median (IQR)	99.53 (89.00-110.55)	98.30 (88.20-109.00)	101.12 (91.03-111.30)	.02
SBP^b^ (mm Hg), median (IQR)	124.00 (112.67-136.67)	120.83 (110.00-131.33)	131.33 (117.33-146.00)	<.001
DBP^c^ (mm Hg), median (IQR)	70.00 (62.09-76.67)	71.33 (65.33-78.00)	66.00 (58.00-74.67)	<.001
Complications and comorbidities, n (%)				
Hypertension				<.001
Yes	3,592,871.3 (48.8)	1,846,042.6 (40.4)	1,746,828.7 (62.5)	
No	3,775,509.2 (51.2)	2,727,516.3 (59.6)	1,047,992.9 (37.5)	
Diabetes				<.001
Yes	1,013,308.4 (13.7)	419,496.7 (9.2)	593,811.8 (21.2)	
No	6,145,988.4 (83.3)	4,033,495.1 (88.2)	2,112,493.3 (75.4)	
Borderline	215,430.4 (2.9)	120,567.2 (2.6)	94,863.3 (3.4)	
Hyperlipidemia				.07
Yes	3,197,057.8 (49.1)	2,076,882.2 (52.3)	1,120,175.6 (44.1)	
No	3,316,009.8 (50.9)	1,895,939.6 (47.7)	1,420,070.1 (55.9)	
Myocardial infarction				<.001
Yes	628,163.1 (8.5)	206,479.8 (4.5)	421,683.3 (15.1)	
No	6,746,564.1 (91.5)	4,367,079.1 (95.5)	2,379,485.0 (84.9)	
Congestive heart failure				<.001
Yes	478,238.4 (6.5)	93,396.5 (2.0)	384,841.9 (13.9)	
No	6,871,854.8 (93.5)	4,480,162.4 (98.0)	2,391,692.4 (86.1)	
Coronary heart disease				<.001
Yes	613,276.9 (8.3)	210,189.8 (4.6)	403,087.2 (14.5)	
No	6,733,110.4 (91.7)	4,358,771.7 (95.4)	2,374,338.8 (85.5)	
Stroke				<.001
Yes	474,510.7 (6.4)	111,899.8 (2.5)	362,610.9 (12.9)	
No	6,891,332.2 (93.6)	4,452,774.8 (97.5)	2,438,557.4 (87.1)	
Weak or failing kidney				<.001
Yes	301,610.5 (4.1)	79,138.2 (1.7)	222,472.3 (7.9)	
No	7,060,022.9 (95.9)	4,481,326.8 (98.3)	2,578,696.0 (92.1)	
Laboratory parameters, median (IQR)				
WBC^d^ (1000 cells/μL)	6.40 (5.30-7.90)	6.20 (5.10-7.80)	6.60 (5.50-8.10)	.01
Neutrophil (1000 cells/μL)	3.80 (3.00-4.90)	3.70 (2.90-4.70)	4.00 (3.20-5.15)	.001
Lymphocyte (1000 cells/μL)	1.70 (1.40-2.20)	1.80 (1.50-2.20)	1.70 (1.20-2.20)	.01
Monocyte (1000 cells/μL)	0.50 (0.40-0.70)	0.50 (0.40-0.60)	0.60 (0.49-0.70)	<.001
RBC^e^ (1,000,000 cells/μL)	4.59 (4.26-4.94)	4.61 (4.32-4.95)	4.56 (4.15-4.89)	.03
Hemoglobin (g/dL)	14.30 (13.40-15.20)	14.30 (13.50-15.20)	14.20 (12.96-15.10)	.07
Platelet (1000 cells/μL)	244.00 (204.00-287.42)	243.00 (207.99-285.46)	245.00 (196.93-293.25)	.55
Albumin (g/dL)	4.20 (4.00-4.40)	4.20 (4.00-4.40)	4.10 (3.90-4.30)	<.001
CRP^f^ (mg/dL)	0.23 (0.09-0.58)	0.18 (0.08-0.47)	0.31 (0.14-0.79)	<.001
SIRI^g^	1.12 (0.76-1.69)	1.01 (0.71-1.48)	1.33 (0.93-2.05)	<.001
SII^h^	528.62 (377.94-745.98)	506.92 (363.83-697.67)	582.50 (403.96-870.95)	<.001
PLR^i^	138.84 (109.59-178.65)	137.88 (109.08-171.82)	141.15 (110.00-189.04)	.10
NLR^j^	2.14 (1.64-3.00)	2.05 (1.59-2.69)	2.36 (1.71-3.44)	<.001
IPI^k^	0.11 (0.04-0.31)	0.09 (0.03-0.23)	0.19 (0.07-0.51)	<.001
CALLY^l^	3.27 (1.22-7.79)	3.97 (1.69-9.83)	2.20 (0.81-5.25)	<.001
Follow-up period (mo), median (IQR)	136.00 (109.00-177.00)	158.00 (130.00-192.50)	89.00 (42.00-131.38)	<.001

a PIR: poverty-to-income ratio.

b SBP: systolic blood pressure.

c DBP: diastolic blood pressure.

d WBC: white blood cell.

e RBC: red blood cell.

f CRP: C-reactive protein.

g SIRI: systemic inflammation response index.

h SII: systemic immune-inflammation index.

i PLR: platelet-to-lymphocyte ratio.

j NLR: neutrophil-to-lymphocyte ratio.

k IPI: inflammatory prognosis index.

l CALLY: C-reactive protein-albumin-lymphocyte index.

### Associations of 6 Inflammatory Markers With Study Outcomes

In Cox regression models, the levels of the 6 inflammatory markers were stratified by tertiles to evaluate their associations with all-cause, cancer-specific, and cardiovascular mortality (Table 2 and Tables S1 and S2 in Multimedia Appendix 1). A 1-SD increase in SIRI was associated with an increased risk of all-cause, cancer-specific, and cardiovascular mortality (all *P*<.001). Similar associations were observed for SII, NLR, and IPI, with elevations in these markers corresponding to higher mortality risks for all 3 outcomes. Associations of PLR and CALLY with all-cause and cardiovascular mortality were observed only in unadjusted or partially adjusted models, with no significant associations with cancer-specific mortality.

**Table 2.** Association between 6 inflammatory markers and all-cause mortality.

	Model I^a^, HR^b^ (95% CI)	*P* value	Model II^c^, HR (95% CI)	*P* value	Model III^d^, HR (95% CI)	*P* value
SIRI^e^						
Per-SD increase	1.40 (1.32-1.49)	<.001	1.28 (1.18-1.39)	<.001	1.32 (1.21-1.45)	<.001
Tertile 1 (≤0.887)	Reference	—^f^	Reference	—	Reference	—
Tertile 2 (>0.887, ≤1.52)	1.97 (1.53-2.54)	<.001	1.60 (1.24-2.06)	<.001	1.65 (1.25-2.17)	<.001
Tertile 3 (>1.52)	2.67 (2.09-3.41)	<.001	1.74 (1.35-2.25)	<.001	1.72 (1.29-2.27)	<.001
*P* value for trend		<.001		<.001		<.001
SII^g^						
Per-SD increase	1.34 (1.24-1.45)	<.001	1.29 (1.18-1.41)	<.001	1.32 (1.19-1.46)	<.001
Tertile 1 (≤429)	Reference	—	Reference	—	Reference	—
Tertile 2 (>429, ≤689)	0.99 (0.78-1.26)	.94	0.85 (0.67-1.09)	.19	0.79 (0.61-1.03)	.09
Tertile 3 (>689)	1.67 (1.34-2.09)	<.001	1.29 (1.02-1.63)	.03	1.16 (0.90-1.49)	.24
*P* value for trend		<.001		.02		.15
PLR^h^						
Per-SD increase	1.23 (1.13-1.35)	<.001	1.14 (1.04-1.26)	.005	1.15 (1.03-1.27)	.01
Tertile 1 (≤119)	Reference	—	Reference	—	Reference	—
Tertile 2 (>119, ≤167)	0.89 (0.71-1.13)	.36	0.80 (0.63-1.01)	.06	0.74 (0.57-0.96)	.02
Tertile 3 (>167)	1.36 (1.09-1.70)	.006	1.09 (0.87-1.36)	.48	1.04 (0.81-1.32)	.78
*P* value for trend		.005		.39		.64
NLR^i^						
Per-SD increase	1.46 (1.35-1.58)	<.001	1.27 (1.16-1.38)	<.001	1.29 (1.17-1.41)	<.001
Tertile 1 (≤1.8)	Reference	—	Reference	—	Reference	—
Tertile 2 (>1.8, ≤2.75)	1.25 (0.98-1.59)	.07	1.05 (0.81-1.35)	.71	1.05 (0.80-1.38)	.72
Tertile 3 (>2.75)	2.05 (1.63-2.59)	<.001	1.44 (1.13-1.83)	.003	1.33 (1.02-1.74)	.03
*P* value for trend		<.001		.002		.02
IPI^j^						
Per-SD increase	1.31 (1.23-1.40)	<.001	1.30 (1.22-1.38)	<.001	1.30 (1.21-1.40)	<.001
Tertile 1 (≤0.072)	Reference	—	Reference	—	Reference	—
Tertile 2 (>0.072, ≤0.237)	1.28 (1.00-1.63)	.046	1.11 (0.87-1.42)	.39	1.18 (0.91-1.54)	.21
Tertile 3 (>0.237)	1.92 (1.53-2.41)	<.001	1.75 (1.38-2.21)	<.001	1.48 (1.14-1.92)	.003
*P* value for trend		<.001		<.001		.003
CALLY^k^						
Per-SD increase	0.79 (0.68-0.92)	.003	0.87 (0.77-0.99)	.04	0.89 (0.78-1.01)	.06
Tertile 1 (≤1.68)	Reference	—	Reference	—	Reference	—
Tertile 2 (>1.68, ≤4.99)	0.72 (0.58-0.89)	.002	0.67 (0.54-0.83)	<.001	0.76 (0.60-0.97)	.03
Tertile 3 (>4.99)	0.55 (0.43-0.69)	<.001	0.59 (0.47-0.75)	<.001	0.66 (0.51-0.85)	.001
*P* value for trend		<.001		<.001		<.001

a Model I was not adjusted for any covariates.

b HR: hazard ratio.

c Model II was adjusted for gender, age, race, educational level, marital status, and poverty-to-income ratio.

d Model III was adjusted for gender, age, race, educational level, marital status, poverty-to-income ratio, smoking, drinking, hypertension, diabetes, hyperlipidemia, congestive heart failure, coronary heart disease, myocardial infarction, and stroke.

e SIRI: systemic inflammation response index.

f Not applicable.

g SII: systemic immune-inflammation index.

h PLR: platelet-to-lymphocyte ratio.

i NLR: neutrophil-to-lymphocyte ratio.

j IPI: inflammatory prognosis index.

k CALLY: C-reactive protein-albumin-lymphocyte index.

With regard to the primary outcome of all-cause mortality, the higher SIRI tertile was associated with an increased risk (tertile 3 vs tertile 1; hazard ratio [HR]: model I: 2.67, 95% CI 2.09‐3.41; model II: 1.74, 95% CI 1.35‐2.25; model III: 1.72, 95% CI 1.29‐2.27). The HRs for NLR and IPI in the second and third tertiles were significantly higher than those in the first (reference) tertile, with *P* value for trend less than .05. CALLY was the only protective factor, with HR values progressively decreasing from tertile 2 to tertile 3 compared with tertile 1 (*P* value for trend<.001).

Regarding cancer-specific mortality, IPI and CALLY appeared to be the most sensitive indicators. A higher IPI level was associated with an increased risk of cancer-specific mortality (tertile 3 vs tertile 1; HR: model I: 2.74, 95% CI 1.74‐4.33; model II: 2.55, 95% CI 1.59‐4.09; model III: 1.91, 95% CI 1.11‐3.27). In contrast, a higher CALLY level was associated with a decreased risk of cancer-specific mortality (tertile 3 vs tertile 1; HR, 95% CI: model I: 0.38, 0.24‐0.60; model II: 0.41, 0.25‐0.65; model III: 0.53, 0.31‐0.90). The other 4 markers were not significantly associated with cancer-specific mortality.

As shown in Table S2 in Multimedia Appendix 1, SIRI may be considered a predictor of cardiovascular mortality. Compared with tertile 1, the HR for tertile 3 was 3.50 (95% CI 1.51‐3.66) in model I, 1.86 (1.06‐3.27) in model II, and 1.83 (0.99‐3.39) in model III, with *P* value for trend of less than .001, .02, and .04, respectively. The other markers were not significantly associated with cardiovascular mortality.

### Dose-Response Relationship of 6 Inflammatory Markers With Study Outcomes

**Figure 1.**
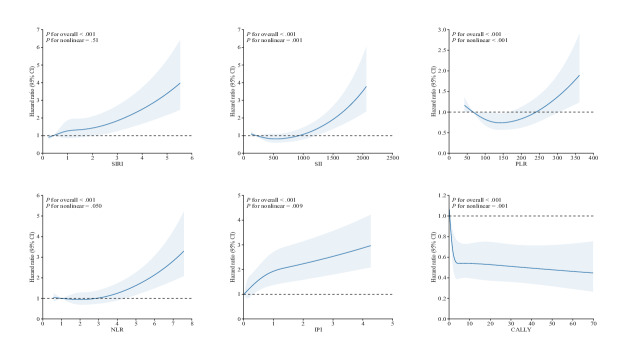
The restricted cubic spline (RCS) curve of the association between SIRI, SII, PLR, NLR, IPI, CALLY, and all-cause mortality. RCS regressions were adjusted for gender, age, race, educational level, marital status, PIR, smoking, drinking, hypertension, diabetes, hyperlipidemia, congestive heart failure, coronary heart disease, myocardial infarction, and stroke. CALLY: C-reactive protein-albumin-lymphocyte index; IPI: inflammatory prognosis index; NLR: neutrophil-to-lymphocyte ratio; PIR: poverty‑to‑income ratio; PLR: platelet-to-lymphocyte ratio; SII: systemic immune-inflammation index; SIRI: systemic inflammation response index.

Multivariable-adjusted RCS analyses revealed significant dose-response relationships between the 6 inflammatory markers and all-cause mortality (Figure 1). SIRI (*P* value for nonlinear=.51) and NLR (*P* value for nonlinear=.05) presented linear correlations, while SII (*P* value for nonlinear=.001), PLR (*P* value for nonlinear<.001), IPI (*P* value for nonlinear=.009), and CALLY (*P* value for nonlinear=.001) exhibited nonlinear associations.

As shown in Figure S3 in Multimedia Appendix 1, SII (*P* value for nonlinear<.001), PLR (*P* value for nonlinear<.001), NLR (*P* value for nonlinear<.001), IPI (*P* value for nonlinear<.001), and CALLY (*P* value for nonlinear=.008) exhibited nonlinear dose-response relationships with cancer-specific mortality, whereas SIRI did not (*P* value for nonlinear=.16). Regarding cardiovascular mortality, SII (*P* value for nonlinear=.005) and CALLY (*P* value for nonlinear=.02) demonstrated nonlinear dose-response relationships, whereas SIRI, NLR, and IPI did not.

### Six Inflammatory Markers as Predictors of the Clinical End Points

**Figure 2.**
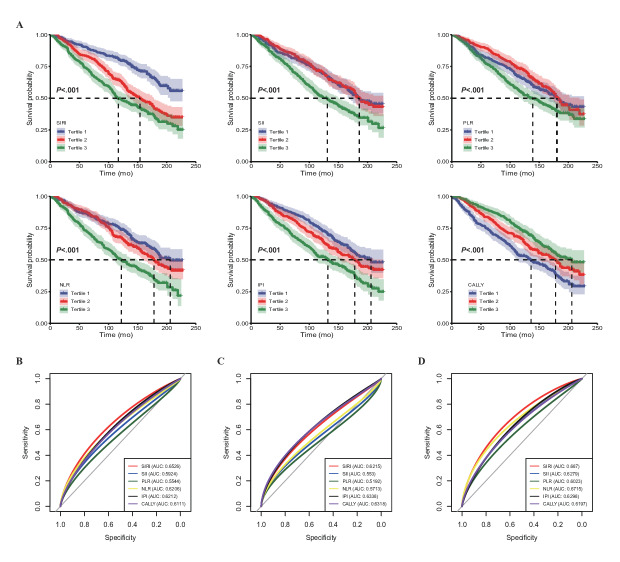
(A) Kaplan-Meier survival curve for all-cause mortality. In the Kaplan-Meier curves, the population is stratified into tertile groups, and statistical analysis is conducted using the log-rank test. (B) Receiver operating characteristic (ROC) curve analysis of SIRI, SII, PLR, NLR, IPI, CALLY, and all-cause mortality. (C) ROC curve analysis of SIRI, SII, PLR, NLR, IPI, CALLY, and cancer mortality. (D) ROC curve analysis of SIRI, SII, PLR, NLR, IPI, CALLY, and cardiovascular mortality. AUC: area under the curve; CALLY: C-reactive protein-albumin-lymphocyte index; IPI: inflammatory prognosis index; NLR: neutrophil-to-lymphocyte ratio; PLR: platelet-to-lymphocyte ratio; SII: systemic immune-inflammation index; SIRI: systemic inflammation response index.

Survival curves for all-cause mortality stratified by tertiles of SIRI, SII, PLR, NLR, IPI, and CALLY are presented in Figure 2A (log-rank test; *P*<.001). Higher levels of SIRI, SII, PLR, NLR, and IPI were associated with worse survival probability, whereas CALLY exhibited the opposite trend.

Notably, the survival trends for cancer-specific and cardiovascular mortality across the 6 inflammatory markers were similar to those observed for all-cause mortality. However, no significant difference in survival probability was observed among PLR tertile groups (Figure S4 in Multimedia Appendix 1).

Figure 2B-D presents the predictive performance of the 6 inflammatory indices for all-cause, cancer-specific, and cardiovascular mortality. For all-cause mortality, the following AUC values were observed: SIRI: 0.6526, SII: 0.5924, PLR: 0.5544, NLR: 0.6206, IPI: 0.6212, and CALLY: 0.6111. Additionally, IPI yielded the highest AUC for cancer-specific mortality, while SIRI was the most efficient predictor of cardiovascular mortality.

### Subgroup Analysis

**Figure 3.**
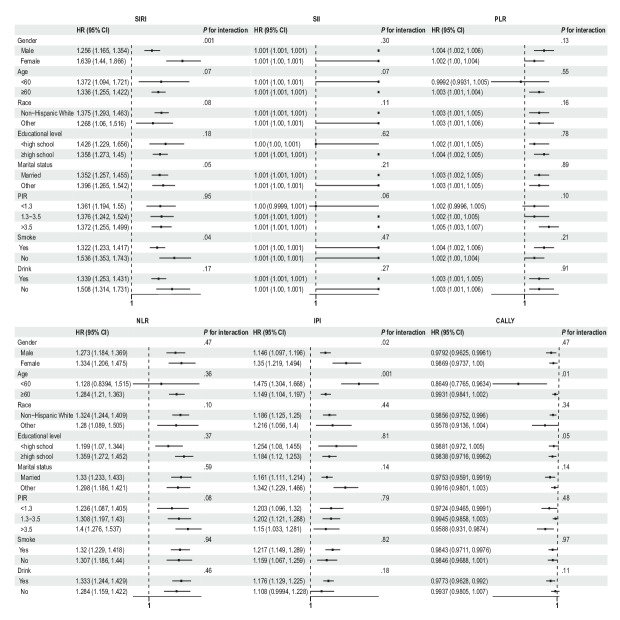
Subgroup analysis for the association between SIRI, SII, PLR, NLR, IPI, CALLY, and all-cause mortality. CALLY: C-reactive protein-albumin-lymphocyte index; HR: hazard ratio; IPI: inflammatory prognosis index; NLR: neutrophil-to-lymphocyte ratio; PIR: poverty-to-income ratio; PLR: platelet-to-lymphocyte ratio; SII: systemic immune-inflammation index; SIRI: systemic inflammation response index.

No significant interactions were observed between SII, PLR, or NLR and all-cause mortality (all *P*>.05). However, gender, age, and smoking were found to potentially interact with SIRI, IPI, and CALLY (Figure 3). Regarding cancer-specific mortality, race, educational level, and marital status exhibited prominent interactions with SIRI, PLR, and IPI (Figure S5 in Multimedia Appendix 1). Furthermore, higher SIRI, SII, and NLR levels were more strongly associated with a higher prevalence of cardiovascular mortality in the married subgroup, whereas a higher cardiovascular mortality risk associated with IPI was observed in the other group (Figure S6 in Multimedia Appendix 1).

### Sensitivity Analysis

After excluding patients with asthma, the risk of all-cause mortality showed a progressively increasing trend across tertiles of SIRI, NLR, and IPI, whereas CALLY showed a progressively decreasing trend (*P* value for trend<.01; Table S3 in Multimedia Appendix 1). After excluding patients with arthritis, the risk of all-cause mortality showed a progressively increasing trend across tertiles of SIRI and IPI, whereas CALLY showed a progressively decreasing trend (*P* value for trend<.05; Table S4 in Multimedia Appendix 1).

## Discussion

To investigate the predictive role of inflammatory biomarkers in patients with cancer, we conducted a cross-sectional study involving 946 participants from the NHANES database. Our findings indicate that SIRI, SII, PLR, NLR, IPI, and CALLY were, to some extent, associated with mortality outcomes in patients with cancer, even after adjustment for selected confounders. Dose-response relationships were observed between most of these inflammatory indicators and mortality outcomes, with the exception of PLR in relation to cardiovascular mortality. Based on receiver operating characteristic curve analyses, the 6 inflammatory biomarkers demonstrated moderate predictive value for all-cause, cancer-specific, and cardiovascular mortality in patients with cancer. Furthermore, subgroup and sensitivity analyses validated the robustness of our models.

It is now well established that inflammation may contribute to the initiation and maintenance of unregulated cellular proliferation, which can lead to tumor formation and growth [9]. Accumulating evidence supports the role of both local immune responses and systemic inflammation in tumor progression and patient survival [23,24]. Systemic inflammatory markers have been linked to increased cancer risk and mortality in numerous studies [25].

SIRI and SII are commonly used to assess the balance between inflammatory response and immune status. Previous studies have found that elevated levels of these markers are associated with poor prognosis in various cancers, including colorectal cancer [26,27], pancreatic cancer [28], breast cancer [29], chordoma [30], and lung cancer [27]. Our study demonstrated that SIRI and SII were elevated in patients who died of cancer, with SIRI showing strong prognostic value across all 3 mortality outcomes. However, the predictive power of SII was less robust in our analysis.

NLR and PLR are major leukocyte-based scores [25] and have been shown to be superior to traditional indicators in renal cell carcinoma [31], multiple sclerosis [32], and acute pancreatitis [33]. In predicting mortality in patients with cancer, NLR may be considered a significant predictor to some extent. Unfortunately, PLR did not demonstrate strong predictive capability in our study.

IPI and CALLY are novel composite indicators that evaluate prognosis through a comprehensive analysis of inflammatory levels, nutritional status, and immune function [17,18]. In our study, IPI was identified as a risk factor for mortality, whereas CALLY was negatively associated with mortality. These findings are consistent with previous cancer-related studies [17,18,34,35]. Both markers are highly promising indicators for predicting prognosis in patients with cancer and warrant further exploration and promotion in future clinical practice.

Collectively, these 6 inflammatory biomarkers—SIRI, SII, PLR, NLR, IPI, and CALLY—represent a simple and economical tool for evaluating systemic inflammation and predicting mortality across multiple cancer types. There is a genuine possibility that these biomarkers may have clinical use in the future. They may enable early stratification of patients for treatment and could potentially be used in clinical settings to determine the optimal timing for treatment modification.

Several limitations of this study should be acknowledged. First, as a single-center, retrospective study, selection bias was inevitable. Therefore, large-scale, multi-institutional, prospective studies are warranted to validate the effectiveness of the examined inflammatory biomarkers. Second, owing to data collection constraints, participants from other survey cycles were not included. In addition, several participants were excluded for failing to meet the inclusion criteria, which may have biased the results. Third, information on cancer history and other diseases was self-reported by participants, rendering it susceptible to recall bias and potential inaccuracies. Moreover, the inability to obtain detailed information on cancer type, stage, or treatment precluded stratified analyses by these variables. This limitation restricts our understanding of the predictive value of inflammatory markers across different malignant tumor types or disease severities. Fourth, this study could not accurately capture the long-term dynamic variations in CBC, CRP, and albumin. Inflammatory markers can vary considerably over time due to infections, treatments, or disease progression. Consequently, future longitudinal studies with repeated biomarker assessments are needed to confirm our findings. Finally, the set of covariates included in this study was incomplete, and some unmeasured confounding factors were not addressed. Specifically, data on the use of anti-inflammatory medications—which are known to lower inflammatory marker levels—were not available. The absence of this information likely introduces nondifferential misclassification of exposure, potentially leading to an underestimation of the true association. Future prospective studies are needed to adjust for these potential confounders.

In conclusion, our preliminary findings indicate that SIRI, NLR, IPI, and CALLY are economical, convenient, and readily available predictors of all-cause, cancer-specific, and cardiovascular mortality in patients with cancer. However, insufficient evidence was found to support the use of SII and PLR as reliable prognostic biomarkers. These inflammatory biomarkers appear to be potential supplementary tools for predicting mortality in patients with cancer. Future prospective trials with long-term follow-up are required to accurately determine the optimal timing and cutoff values that may be applied in clinical practice.

## Supplementary material

10.2196/99410 Multimedia Appendix 1 Supplemental figures and tables for inflammatory markers and mortality outcomes.

## Abbreviations

AUC: area under the curve
CALLY: C-reactive protein-albumin-lymphocyte
CBC: complete blood count
CHD: coronary heart disease
CHF: congestive heart failure
CRP: C-reactive protein
HR: hazard ratio
*ICD-10*: *International Classification of Diseases, 10th Revision*
IPI: inflammatory prognostic index
MI: myocardial infarction
NHANES: National Health and Nutrition Examination Survey
NLR: neutrophil-to-lymphocyte ratio
PLR: platelet-to-lymphocyte ratio
RCS: restricted cubic spline
SII: systemic immune-inflammation index
SIRI: systemic inflammatory response index
