# Supplementary material for: Clinical Feasibility of Six Inflammatory Markers for Predicting the Mortality of Patients With Cancer: Longitudinal Study
Source: JMIR Cancer. 2026 Jul 22;12:e99410. doi: 10.2196/99410 (PMC13390893; doi:10.2196/99410)
Supplement: Multimedia Appendix 1 [file cancer-v12-e99410-s001.docx]

 **Supplementary Figure 1:** Flow chat of the study population. NHANES, National Health and Nutrition Examination Survey; CRP, C-reactive protein; PIR, poverty-to-income ratio.


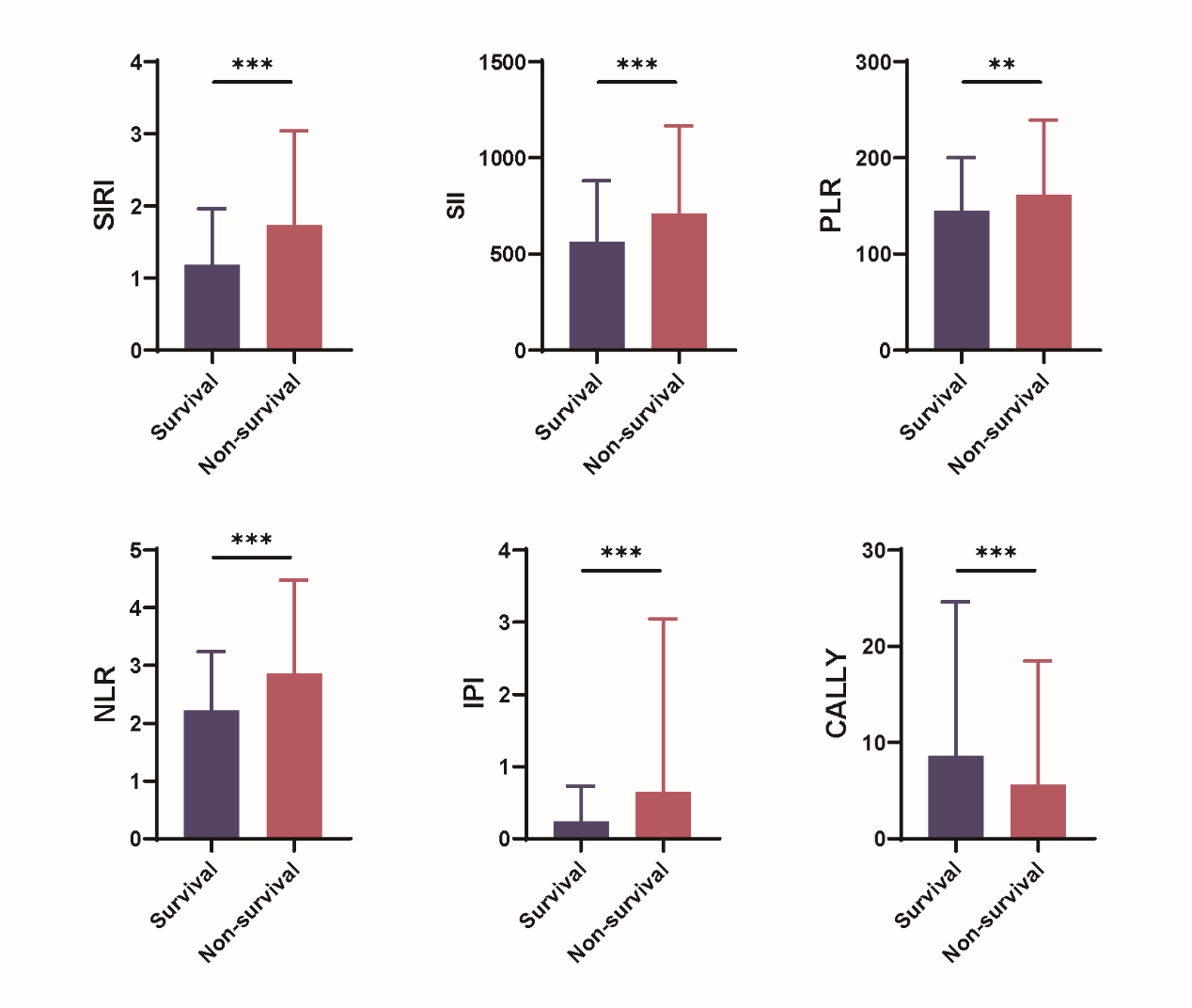


**Supplementary Figure 2:** Differences in SIRI, SII, PLR, NLR, IPI and CALLY between survival group and non-survival group. SIRI, systemic inflammation response index; SII, systemic immune-inflammation index; PLR, platelet-to-lymphocyte ratio; NLR, neutrophil-to-lymphocyte ratio; IPI, inflammatory prognosis index; CALLY, C-reactive protein-albumin-lymphocyte. **P < 0.01; ***P< 0.001.

**
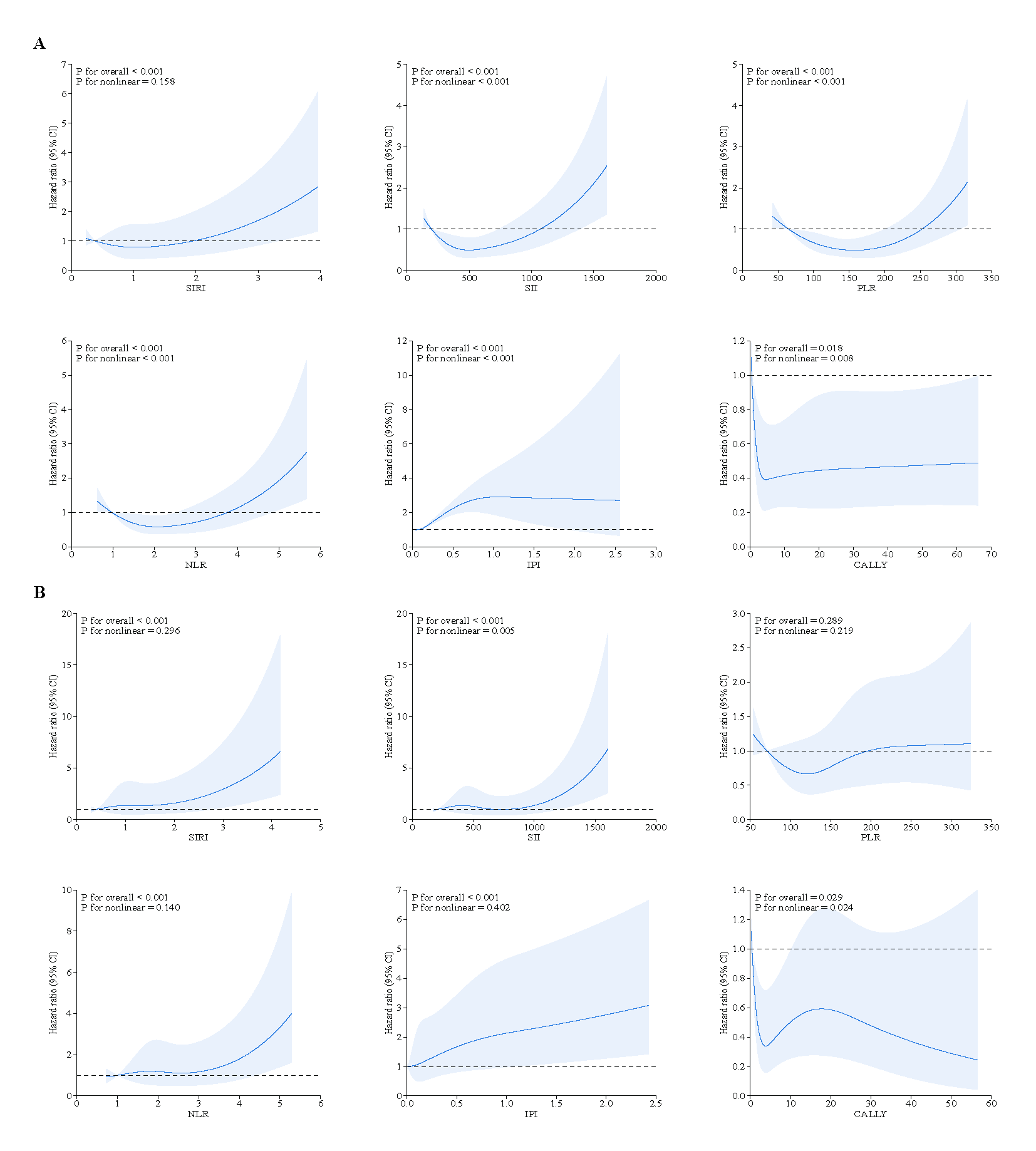
****Supplementary** **Figure 3:** (A) The RCS curve of the association between SIRI, SII, PLR, NLR, IPI, CALLY and cancer mortality. (B) The RCS curve of the association between SIRI, SII, PLR, NLR, IPI, CALLY and cardiovascular mortality. RCS regressions were adjusted for gender, age, race, educational level, marital status, PIR, smoking, drinking, hypertension, diabetes, hyperlipemia, congestive heart failure, coronary heart disease, myocardial infarction and stroke. SIRI, systemic inflammation response index; SII, systemic immune-inflammation index; PLR, platelet-to-lymphocyte ratio; NLR, neutrophil-to-lymphocyte ratio; IPI, inflammatory prognosis index; CALLY, C-reactive protein-albumin-lymphocyte.

**
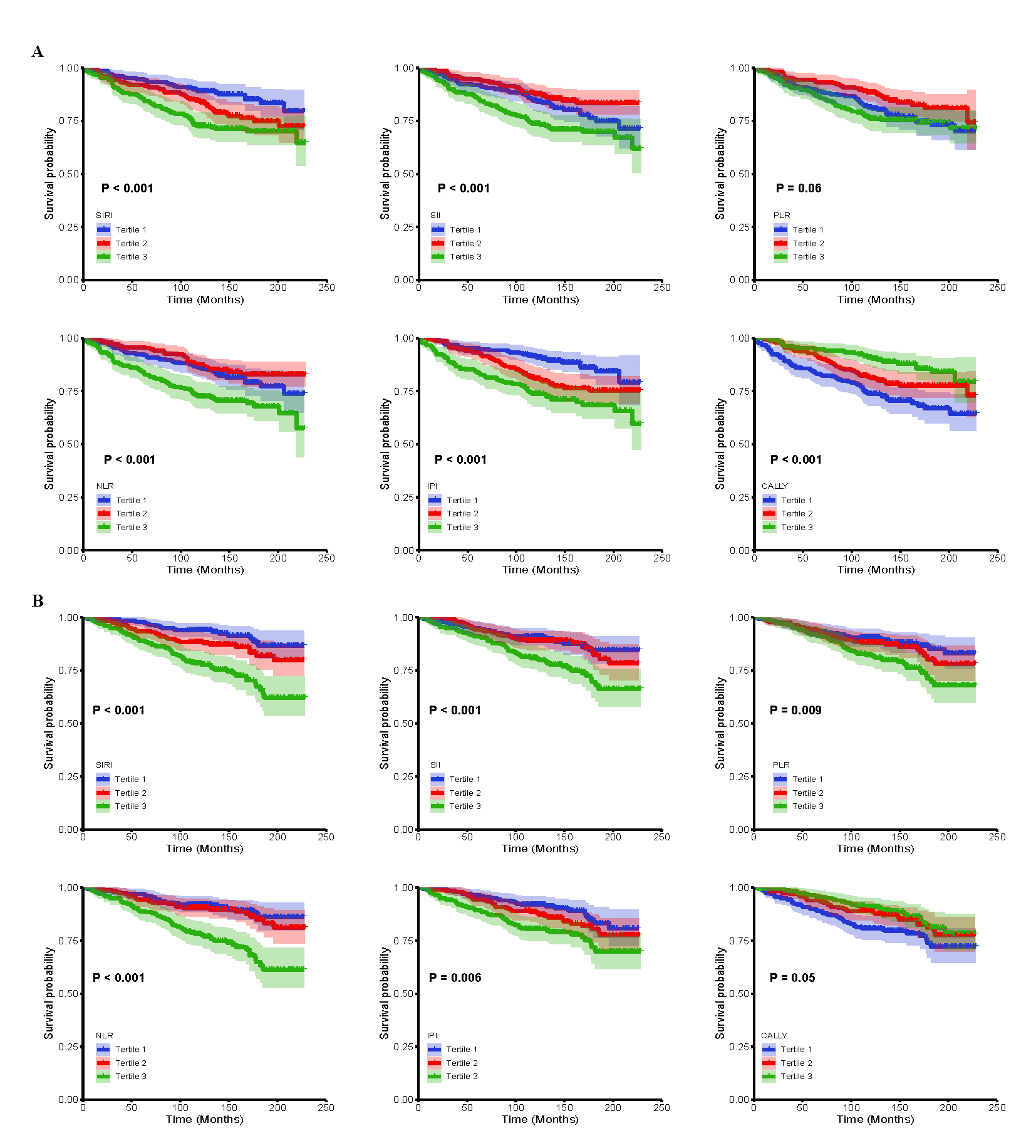
Supplementary Figure 4:** (A) Kaplan-Meier survival curve for cancer mortality. (B) Kaplan-Meier survival curve for cardiovascular mortality. In the Kaplan-Meier curves, the population is stratified into tertile groups, and statistical analysis is conducted using the log-rank test. SIRI, systemic inflammation response index; SII, systemic immune-inflammation index; PLR, platelet-to-lymphocyte ratio; NLR, neutrophil-to-lymphocyte ratio; IPI, inflammatory prognosis index; CALLY, C-reactive protein-albumin-lymphocyte.

**
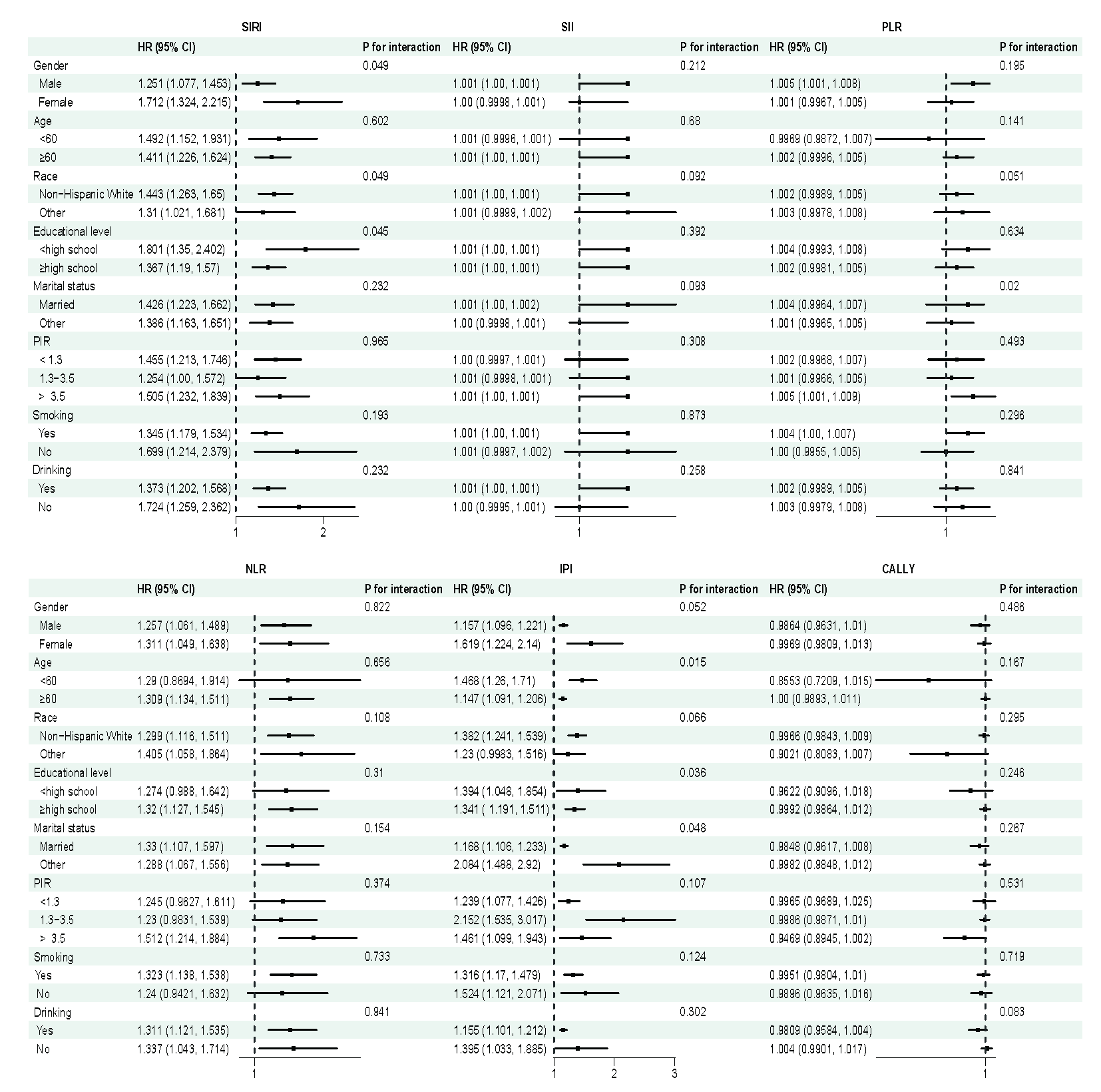
Supplementary Figure 5:** Subgroup analysis for the association between SIRI, SII, PLR, NLR, IPI, CALLY and cancer mortality. SIRI, systemic inflammation response index; SII, systemic immune-inflammation index; PLR, platelet-to-lymphocyte ratio; NLR, neutrophil-to-lymphocyte ratio; IPI, inflammatory prognosis index; CALLY, C-reactive protein-albumin-lymphocyte; PIR, poverty-to-income ratio; HR, hazard ratio; CI, confidence interval.

**
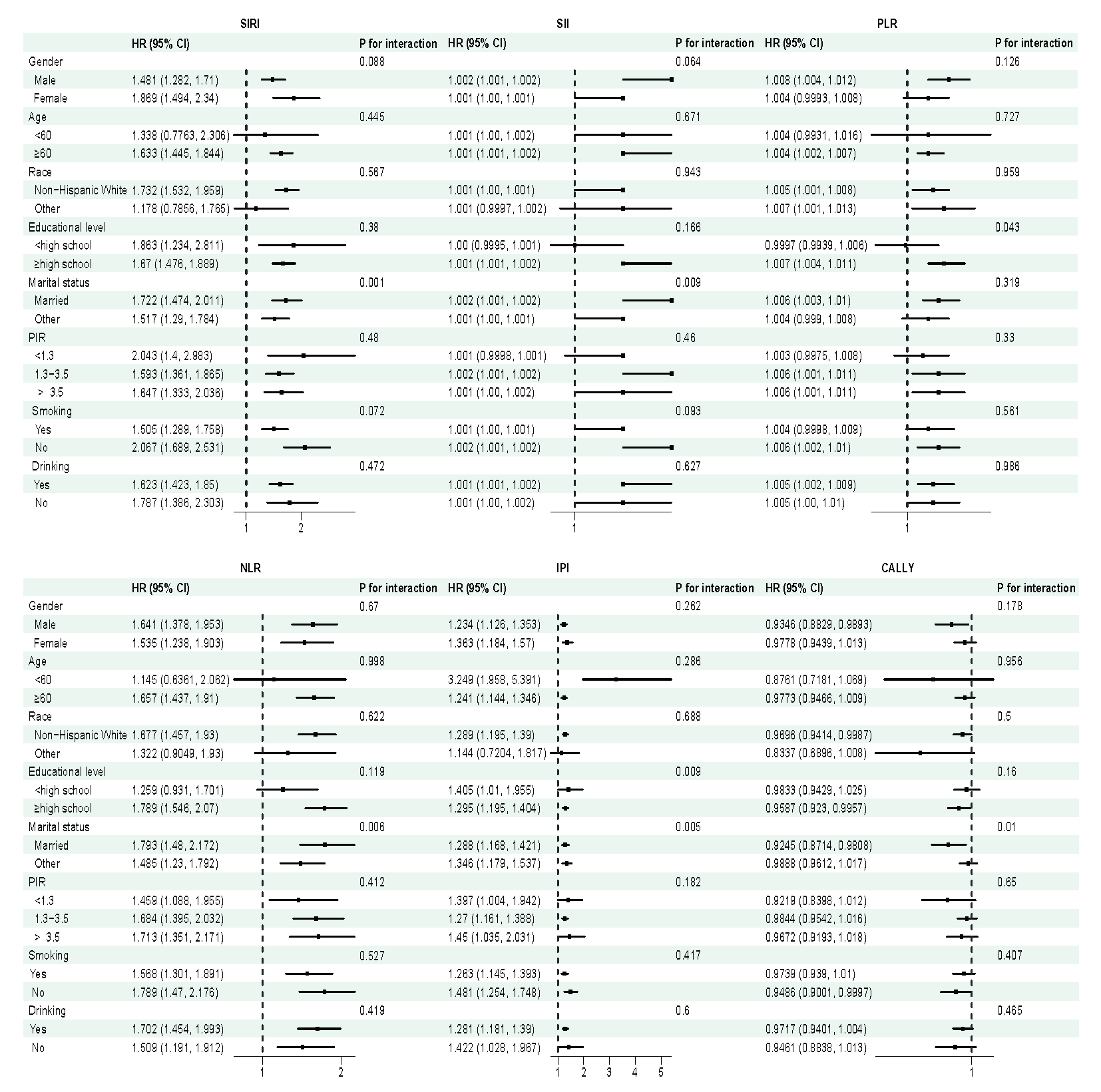
Supplementary Figure 6:** Subgroup analysis for the association between SIRI, SII, PLR, NLR, IPI, CALLY and cardiovascular mortality. SIRI, systemic inflammation response index; SII, systemic immune-inflammation index; PLR, platelet-to-lymphocyte ratio; NLR, neutrophil-to-lymphocyte ratio; IPI, inflammatory prognosis index; CALLY, C-reactive protein-albumin-lymphocyte; PIR, poverty-to-income ratio; HR, hazard ratio; CI, confidence interval.

Supplementary Table 1: The association between six inflammatory markers and cancer mortality.

|  | Model I  HR (95% CI) | P-value | Model II  HR (95% CI) | P-value | Model III  HR (95% CI) | P-value |
| --- | --- | --- | --- | --- | --- | --- |
| SIRI |  |  |  |  |  |  |
| Per-SD increase | 1.37 (1.23, 1.52) | <0.001 | 1.36 (1.18, 1.56) | <0.001 | 1.42 (1.19, 1.70) | <0.001 |
| Tertile 1 (≦0.82) | Reference |  | Reference |  | Reference |  |
| Tertile 2 (>0.82, ≦1.36) | 1.64 (1.03, 2.61) | 0.037 | 1.39 (0.86, 2.23) | 0.178 | 1.47 (0.87, 2.50) | 0.106 |
| Tertile 3 (>1.36) | 2.35 (1.51, 3.66) | <0.001 | 1.72 (1.09, 2.72) | 0.019 | 1.63 (0.95, 2.80) | 0.092 |
| P for trend |  | <0.001 |  | 0.019 |  | 0.103 |
| SII |  |  |  |  |  |  |
| Per-SD increase | 1.25 (1.10, 1.43) | <0.001 | 1.32 (1.13, 1.54) | <0.001 | 1.33 (1.10, 1.61) | 0.003 |
| Tertile 1 (≦411) | Reference |  | Reference |  | Reference |  |
| Tertile 2 (>411, ≦633) | 0.70 (0.44, 1.11) | 0.129 | 0.71 (0.44, 1.13) | 0.15 | 0.65 (0.39, 1.10) | 0.106 |
| Tertile 3 (>633) | 1.54 (1.05, 2.28) | 0.029 | 1.56 (1.05, 2.33) | 0.029 | 1.26 (0.80, 1.99) | 0.326 |
| P for trend |  | 0.02 |  | 0.018 |  | 0.276 |
| PLR |  |  |  |  |  |  |
| Per-SD increase | 1.16 (0.98, 1.36) | 0.089 | 1.15 (0.97, 1.36) | 0.114 | 1.11 (0.91, 1.35) | 0.294 |
| Tertile 1 (≦116) | Reference |  | Reference |  | Reference |  |
| Tertile 2 (>116, ≦162) | 0.66 (0.43, 1.03) | 0.067 | 0.64 (0.41, 1.00) | 0.052 | 0.66 (0.40, 1.09) | 0.103 |
| Tertile 3 (>162) | 1.10 (0.75, 1.63) | 0.619 | 1.07 (0.72, 1.60) | 0.742 | 0.94 (0.60, 1.47) | 0.776 |
| P for trend |  | 0.604 |  | 0.729 |  | 0.736 |
| NLR |  |  |  |  |  |  |
| Per-SD increase | 1.37 (1.18, 1.59) | <0.001 | 1.28 (1.09, 1.50) | 0.002 | 1.30 (1.08, 1.56) | 0.006 |
| Tertile 1 (≦1.73) | Reference |  | Reference |  | Reference |  |
| Tertile 2 (>1.73, ≦2.53) | 0.79 (0.50, 1.26) | 0.323 | 0.79 (0.49, 1.27) | 0.323 | 0.78 (0.46, 1.32) | 0.349 |
| Tertile 3 (>2.53) | 1.79 (1.21, 2.66) | 0.004 | 1.45 (0.96, 2.19) | 0.081 | 1.26 (0.79, 2.02) | 0.333 |
| P for trend |  | 0.002 |  | 0.048 |  | 0.253 |
| IPI |  |  |  |  |  |  |
| Per-SD increase | 1.33 (1.22, 1.45) | <0.001 | 1.30 (1.19, 1.42) | <0.001 | 1.34 (1.19, 1.52) | <0.001 |
| Tertile 1 (≦0.0669) | Reference |  | Reference |  | Reference |  |
| Tertile 2 (>0.0669, ≦0.212) | 1.86 (1.15, 3.00) | 0.011 | 1.60 (0.98, 2.63) | 0.062 | 1.54 (0.90, 2.65) | 0.116 |
| Tertile 3 (>0.212) | 2.74 (1.74, 4.33) | <0.001 | 2.55 (1.59, 4.09) | <0.001 | 1.91 (1.11, 3.27) | 0.019 |
| P for trend |  | <0.001 |  | <0.001 |  | 0.02 |
| CALLY |  |  |  |  |  |  |
| Per-SD increase | 0.89 (0.71, 1.11) | 0.305 | 0.92 (0.77, 1.11) | 0.377 | 0.95 (0.80, 1.13) | 0.559 |
| Tertile 1 (≦1.84) | Reference |  | Reference |  | Reference |  |
| Tertile 2 (>1.84, ≦5.38) | 0.66 (0.45, 0.97) | 0.032 | 0.64 (0.43, 0.95) | 0.027 | 0.79 (0.51, 1.24) | 0.306 |
| Tertile 3 (>5.38) | 0.38 (0.24, 0.60) | <0.001 | 0.41 (0.25, 0.65) | <0.001 | 0.53 (0.31, 0.90) | 0.02 |
| P for trend |  | <0.001 |  | <0.001 |  | 0.02 |

SIRI, systemic inflammation response index; SII, systemic immune-inflammation index; PLR, platelet-to-lymphocyte ratio; NLR, neutrophil-to-lymphocyte ratio; IPI, inflammatory prognosis index; CALLY, C-reactive protein-albumin-lymphocyte; SD, standard deviation; HR, hazard ratio; CI, confidence interval.

Model I was not adjusted for any covariates.

Model II was adjusted for gender, age, race, educational level, marital status and PIR.

Model III was adjusted for gender, age, race, educational level, marital status, PIR, smoking, drinking, hypertension, diabetes, hyperlipemia, congestive heart failure, coronary heart disease, myocardial infarction and stroke.

Supplementary Table 2: The association between six inflammatory markers and cardiovascular mortality.

|  | Model I  HR (95% CI) | P-value | Model II  HR (95% CI) | P-value | Model III  HR (95% CI) | P-value |
| --- | --- | --- | --- | --- | --- | --- |
| SIRI |  |  |  |  |  |  |
| Per-SD increase | 1.59 (1.43, 1.77) | <0.001 | 1.50 (1.28, 1.75) | <0.001 | 1.68 (1.39, 2.02) | <0.001 |
| Tertile 1 (≦0.821) | Reference |  | Reference |  | Reference |  |
| Tertile 2 (>0.821, ≦1.4) | 1.72 (0.96, 3.09) | 0.069 | 1.34 (0.74, 2.43) | 0.332 | 1.28 (0.67, 2.45) | 0.448 |
| Tertile 3 (>1.4) | 3.50 (1.51, 3.66) | <0.001 | 1.86 (1.06, 3.27) | 0.031 | 1.83 (0.99, 3.39) | 0.054 |
| P for trend |  | <0.001 |  | 0.024 |  | 0.041 |
| SII |  |  |  |  |  |  |
| Per-SD increase | 1.42 (1.25, 1.61) | <0.001 | 1.44 (1.19, 1.74) | <0.001 | 1.48 (1.20, 1.84) | <0.001 |
| Tertile 1 (≦418) | Reference |  | Reference |  | Reference |  |
| Tertile 2 (>418, ≦635) | 1.18 (0.69, 2.03) | 0.549 | 0.99 (0.57, 1.74) | 0.983 | 1.02 (0.54, 1.92) | 0.956 |
| Tertile 3 (>635) | 2.25 (1.39, 3.65) | 0.001 | 1.48 (0.88, 2.47) | 0.136 | 1.30 (0.73, 2.29) | 0.373 |
| P for trend |  | <0.001 |  | 0.098 |  | 0.325 |
| PLR |  |  |  |  |  |  |
| Per-SD increase | 1.36 (1.15, 1.60) | <0.001 | 1.18 (0.98, 1.41) | 0.088 | 1.11 (0.90, 1.36) | 0.333 |
| Tertile 1 (≦119) | Reference |  | Reference |  | Reference |  |
| Tertile 2 (>119, ≦163) | 1.27 (0.75, 2.14) | 0.382 | 1.03 (0.59, 1.80) | 0.907 | 0.85 (0.46, 1.57) | 0.608 |
| Tertile 3 (>163) | 2.01 (1.24, 3.25) | 0.005 | 1.38 (0.84, 2.28) | 0.207 | 1.24 (0.72, 2.14) | 0.432 |
| P for trend |  | 0.003 |  | 0.168 |  | 0.315 |
| NLR |  |  |  |  |  |  |
| Per-SD increase | 1.77 (1.52, 2.06) | <0.001 | 1.43 (1.19, 1.71) | <0.001 | 1.47 (1.18, 1.82) | <0.001 |
| Tertile 1 (≦1.75) | Reference |  | Reference |  | Reference |  |
| Tertile 2 (>1.75, ≦2.58) | 1.24 (0.69, 2.20) | 0.472 | 1.03 (0.57, 1.86) | 0.935 | 1.26 (0.65, 2.46) | 0.49 |
| Tertile 3 (>2.58) | 3.08 (1.87, 5.07) | <0.001 | 1.58 (0.92, 2.71) | 0.096 | 1.72 (0.92, 3.21) | 0.092 |
| P for trend |  | <0.001 |  | 0.057 |  | 0.078 |
| IPI |  |  |  |  |  |  |
| Per-SD increase | 1.30 (1.20, 1.40) | <0.001 | 1.28 (1.18, 1.39) | <0.001 | 1.34 (1.21, 1.47) | <0.001 |
| Tertile 1 (≦0.0604) | Reference |  | Reference |  | Reference |  |
| Tertile 2 (>0.0604, ≦0.207) | 1.40 (0.83, 2.35) | 0.212 | 0.99 (0.57, 1.72) | 0.977 | 0.95 (0.51, 1.74) | 0.86 |
| Tertile 3 (>0.207) | 2.14 (1.31, 3.50) | 0.002 | 2.04 (1.22, 3.41) | 0.007 | 1.41 (0.79, 2.51) | 0.245 |
| P for trend |  | 0.002 |  | 0.003 |  | 0.216 |
| CALLY |  |  |  |  |  |  |
| Per-SD increase | 0.58 (0.36, 0.91) | 0.019 | 0.69 (0.42, 1.14) | 0.145 | 0.77 (0.53, 1.14) | 0.191 |
| Tertile 1 (≦1.95) | Reference |  | Reference |  | Reference |  |
| Tertile 2 (>1.95, ≦5.75) | 0.67 (0.43, 1.06) | 0.087 | 0.46 (0.29, 0.75) | 0.002 | 0.58 (0.32, 1.04) | 0.068 |
| Tertile 3 (>5.75) | 0.58 (0.36, 0.92) | 0.022 | 0.57 (0.34, 0.93) | 0.024 | 0.77 (0.43, 1.38) | 0.384 |
| P for trend |  | 0.019 |  | 0.016 |  | 0.374 |

SIRI, systemic inflammation response index; SII, systemic immune-inflammation index; PLR, platelet-to-lymphocyte ratio; NLR, neutrophil-to-lymphocyte ratio; IPI, inflammatory prognosis index; CALLY, C-reactive protein-albumin-lymphocyte; SD, standard deviation; HR, hazard ratio; CI, confidence interval.

Model I was not adjusted for any covariates.

Model II was adjusted for gender, age, race, educational level, marital status and PIR.

Model III was adjusted for gender, age, race, educational level, marital status, PIR, smoking, drinking, hypertension, diabetes, hyperlipemia, congestive heart failure, coronary heart disease, myocardial infarction and stroke.

Supplementary Table 3: The association between six inflammatory markers and all-cause mortality after excluding patients with asthma.

|  | Model I  HR (95% CI) | P-value | Model II  HR (95% CI) | P-value | Model III  HR (95% CI) | P-value |
| --- | --- | --- | --- | --- | --- | --- |
| SIRI |  |  |  |  |  |  |
| Per-SD increase | 1.40 (1.31, 1.50) | <0.001 | 1.27 (1.16, 1.38) | <0.001 | 1.30 (1.18, 1.42) | <0.001 |
| Tertile 1 (≦0.891) | Reference |  | Reference |  | Reference |  |
| Tertile 2 (>0.891, ≦1.53) | 1.91 (1.47, 2.48) | <0.001 | 1.51 (1.15, 1.97) | 0.003 | 1.54 (1.16, 2.06) | 0.003 |
| Tertile 3 (>1.53) | 2.71 (2.10, 3.49) | <0.001 | 1.74 (1.34, 2.27) | <0.001 | 1.70 (1.27, 2.27) | <0.001 |
| P for trend |  | <0.001 |  | <0.001 |  | <0.001 |
| SII |  |  |  |  |  |  |
| Per-SD increase | 1.39 (1.28, 1.52) | <0.001 | 1.29 (1.18, 1.42) | <0.001 | 1.29 (1.16, 1.44) | <0.001 |
| Tertile 1 (≦427) | Reference |  | Reference |  | Reference |  |
| Tertile 2 (>427, ≦687) | 0.95 (0.74, 1.22) | 0.671 | 0.85 (0.66, 1.10) | 0.216 | 0.84 (0.64, 1.10) | 0.215 |
| Tertile 3 (>687) | 1.71 (1.35, 2.15) | <0.001 | 1.27 (1.01, 1.62) | 0.044 | 1.15 (0.89, 1.49) | 0.296 |
| P for trend |  | <0.001 |  | 0.029 |  | 0.222 |
| PLR |  |  |  |  |  |  |
| Per-SD increase | 1.26 (1.15, 1.38) | <0.001 | 1.16 (1.05, 1.27) | 0.003 | 1.13 (1.02, 1.26) | 0.022 |
| Tertile 1 (≦120) | Reference |  | Reference |  | Reference |  |
| Tertile 2 (>120 ≦167) | 0.84 (0.66, 1.08) | 0.180 | 0.78 (0.61, 1.00) | 0.049 | 0.71 (0.55, 0.94) | 0.014 |
| Tertile 3 (>167) | 1.39 (1.11, 1.74) | 0.005 | 1.09 (0.87, 1.36) | 0.404 | 1.04 (0.81, 1.34) | 0.746 |
| P for trend |  | 0.004 |  | 0.327 |  | 0.603 |
| NLR |  |  |  |  |  |  |
| Per-SD increase | 1.48 (1.37, 1.61) | <0.001 | 1.26 (1.15, 1.38) | <0.001 | 1.27 (1.16, 1.40) | <0.001 |
| Tertile 1 (≦1.81) | Reference |  | Reference |  | Reference |  |
| Tertile 2 (>1.81, ≦2.77) | 1.29 (1.00, 1.66) | 0.052 | 1.04 (0.80, 1.35) | 0.790 | 1.06 (0.80, 1.40) | 0.708 |
| Tertile 3 (>2.77) | 2.18 (1.72, 2.77) | <0.001 | 1.51 (1.18, 1.93) | 0.001 | 1.41 (1.07, 1.85) | 0.013 |
| P for trend |  | <0.001 |  | <0.001 |  | 0.009 |
| IPI |  |  |  |  |  |  |
| Per-SD increase | 1.31 (1.23, 1.41) | <0.001 | 1.30 (1.21, 1.39) | <0.001 | 1.29 (1.19, 1.39) | <0.001 |
| Tertile 1 (≦0.0691) | Reference |  | Reference |  | Reference |  |
| Tertile 2 (>0.0691, ≦0.23) | 1.24 (0.96, 1.59) | 0.097 | 1.10 (0.86, 1.42) | 0.450 | 1.12 (0.85, 1.47) | 0.431 |
| Tertile 3 (>0.23) | 1.95 (1.54, 2.47) | <0.001 | 1.72 (1.35, 2.19) | <0.001 | 1.52 (1.17, 1.98) | 0.002 |
| P for trend |  | <0.001 |  | <0.001 |  | 0.002 |
| CALLY |  |  |  |  |  |  |
| Per-SD increase | 0.79 (0.67, 0.92) | 0.003 | 0.86 (0.75, 0.99) | 0.034 | 0.88 (0.77, 1.01) | 0.073 |
| Tertile 1 (≦1.79) | Reference |  | Reference |  | Reference |  |
| Tertile 2 (>1.79, ≦5.21) | 0.70 (0.56, 0.87) | 0.002 | 0.70 (0.55, 0.87) | 0.002 | 0.75 (0.59, 0.96) | 0.022 |
| Tertile 3 (>5.21) | 0.54 (0.42, 0.68) | <0.001 | 0.59 (0.46, 0.75) | <0.001 | 0.65 (0.50, 0.85) | 0.002 |
| P for trend |  | <0.001 |  | <0.001 |  | 0.001 |

SIRI, systemic inflammation response index; SII, systemic immune-inflammation index; PLR, platelet-to-lymphocyte ratio; NLR, neutrophil-to-lymphocyte ratio; IPI, inflammatory prognosis index; CALLY, C-reactive protein-albumin-lymphocyte; SD, standard deviation; HR, hazard ratio; CI, confidence interval.

Model I was not adjusted for any covariates.

Model II was adjusted for gender, age, race, educational level, marital status and PIR.

Model III was adjusted for gender, age, race, educational level, marital status, PIR, smoking, drinking, hypertension, diabetes, hyperlipemia, congestive heart failure, coronary heart disease, myocardial infarction and stroke.

Supplementary Table 4: The association between six inflammatory markers and all-cause mortality after excluding patients with arthritis.

|  | Model I  HR (95% CI) | P-value | Model II  HR (95% CI) | P-value | Model III  HR (95% CI) | P-value |
| --- | --- | --- | --- | --- | --- | --- |
| SIRI |  |  |  |  |  |  |
| Per-SD increase | 1.30 (1.18, 1.42) | <0.001 | 1.15 (1.02, 1.30) | 0.023 | 1.23 (1.08, 1.41) | 0.003 |
| Tertile 1 (≦0.84) | Reference |  | Reference |  | Reference |  |
| Tertile 2 (>0.84, ≦1.4) | 1.36 (0.95, 1.97) | 0.097 | 1.26 (0.87, 1.82) | 0.218 | 1.48 (0.99, 2.22) | 0.056 |
| Tertile 3 (>1.4) | 2.32 (1.65, 3.26) | <0.001 | 1.44 (1.01, 2.05) | 0.045 | 1.57 (1.04, 2.37) | 0.031 |
| P for trend |  | <0.001 |  | 0.046 |  | 0.036 |
| SII |  |  |  |  |  |  |
| Per-SD increase | 1.22 (1.09, 1.37) | <0.001 | 1.19 (1.05, 1.36) | 0.009 | 1.23 (1.06, 1.43) | 0.007 |
| Tertile 1 (≦416) | Reference |  | Reference |  | Reference |  |
| Tertile 2 (>416, ≦673) | 0.86 (0.60, 1.22) | 0.395 | 0.84 (0.59, 1.20) | 0.347 | 0.82 (0.55, 1.21) | 0.318 |
| Tertile 3 (>673) | 1.51 (1.09, 2.08) | 0.012 | 1.22 (0.88, 1.69) | 0.238 | 1.07 (0.74, 1.55) | 0.731 |
| P for trend |  | 0.009 |  | 0.206 |  | 0.641 |
| PLR |  |  |  |  |  |  |
| Per-SD increase | 1.12 (0.98, 1.28) | 0.094 | 1.06 (0.92, 1.21) | 0.428 | 1.08 (0.93, 1.26) | 0.308 |
| Tertile 1 (≦121) | Reference |  | Reference |  | Reference |  |
| Tertile 2 (>121, ≦167) | 1.03 (0.73, 1.45) | 0.869 | 0.95 (0.67, 1.35) | 0.782 | 0.81 (0.55, 1.20) | 0.292 |
| Tertile 3 (>167) | 1.26 (0.90, 1.74) | 0.175 | 1.06 (0.76, 1.48) | 0.739 | 0.98 (0.68, 1.40) | 0.911 |
| P for trend |  | 0.170 |  | 0.714 |  | 0.980 |
| NLR |  |  |  |  |  |  |
| Per-SD increase | 1.28 (1.14, 1.43) | <0.001 | 1.08 (0.96 1.23) | 0.203 | 1.12 (0.97, 1.28) | 0.118 |
| Tertile 1 (≦1.79) | Reference |  | Reference |  | Reference |  |
| Tertile 2 (>1.79, ≦2.68) | 1.30 (0.92, 1.86) | 0.141 | 1.10 (0.76, 1.57) | 0.618 | 1.13 (0.76, 1.68) | 0.551 |
| Tertile 3 (>2.68) | 1.83 (1.31, 2.56) | <0.001 | 1.18 (0.83, 1.67) | 0.352 | 1.15 (0.78, 1.69) | 0.481 |
| P for trend |  | <0.001 |  | 0.351 |  | 0.500 |
| IPI |  |  |  |  |  |  |
| Per-SD increase | 1.13 (1.05, 1.22) | 0.002 | 1.12 (1.04, 1.21) | 0.004 | 1.34 (1.21, 1.47) | 0.012 |
| Tertile 1 (≦0.0562) | Reference |  | Reference |  | Reference |  |
| Tertile 2 (>0.0562, ≦0.2) | 1.64 (1.15, 2.36) | 0.007 | 1.25 (0.87, 1.80) | 0.233 | 1.25 (0.83, 1.87) | 0.286 |
| Tertile 3 (>0.2) | 2.22 (1.57, 3.15) | <0.001 | 1.91 (1.34, 2.73) | <0.001 | 1.70 (1.13, 2.56) | 0.012 |
| P for trend |  | <0.001 |  | <0.001 |  | 0.010 |
| CALLY |  |  |  |  |  |  |
| Per-SD increase | 0.60 (0.44, 0.80) | <0.001 | 0.75 (0.56, 1.00) | 0.048 | 0.79 (0.59, 1.05) | 0.101 |
| Tertile 1 (≦2.05) | Reference |  | Reference |  | Reference |  |
| Tertile 2 (>2.05, ≦6.31) | 0.74 (0.54, 1.01) | 0.056 | 0.75 (0.55, 1.03) | 0.078 | 0.83 (0.58, 1.18) | 0.293 |
| Tertile 3 (>6.31) | 0.48 (0.34, 0.67) | <0.001 | 0.57 (0.40, 0.80) | 0.001 | 0.59 (0.40, 0.88) | 0.009 |
| P for trend |  | <0.001 |  | 0.001 |  | 0.009 |

SIRI, systemic inflammation response index; SII, systemic immune-inflammation index; PLR, platelet-to-lymphocyte ratio; NLR, neutrophil-to-lymphocyte ratio; IPI, inflammatory prognosis index; CALLY, C-reactive protein-albumin-lymphocyte; SD, standard deviation; HR, hazard ratio; CI, confidence interval.

Model I was not adjusted for any covariates.

Model II was adjusted for gender, age, race, educational level, marital status and PIR.

Model III was adjusted for gender, age, race, educational level, marital status, PIR, smoking, drinking, hypertension, diabetes, hyperlipemia, congestive heart failure, coronary heart disease, myocardial infarction and stroke.
